# Characterization of Starch Degradation Related Genes in Postharvest Kiwifruit

**DOI:** 10.3390/ijms17122112

**Published:** 2016-12-15

**Authors:** Xiong Hu, Sheng Kuang, Ai-Di Zhang, Wang-Shu Zhang, Miao-Jin Chen, Xue-Ren Yin, Kun-Song Chen

**Affiliations:** 1 Zhejiang Provincial Key Laboratory of Horticultural Plant Integrative Biology, Zhejiang University, Zijingang Campus, Hangzhou 310058, China; huxiongzju@163.com (X.H.); 21416046@zju.edu.cn (S.K.); aidi2012@126.com (A.-D.Z.); akun@zju.edu.cn (K.-S.C.); 2 The State Agriculture Ministry Laboratory of Horticultural Plant Growth, Development and Quality Improvement, Zhejiang University, Zijingang Campus, Hangzhou 310058, China; 3 Ningbo Fullharvest Agriculture Technology Co., Ltd., Ningbo 315202, China; zjuzws@zju.edu.cn; 4 Fenghua Peach Research Institute, Ningbo 315502, China; mjchen04@163.com

**Keywords:** kiwifruit, postharvest ripening, starch degradation, amylase

## Abstract

Starch is one of the most important storage carbohydrates in plants. Kiwifruit typically accumulate large amounts of starch during development. The fruit retain starch until commercial maturity, and its postharvest degradation is essential for consumer acceptance. The activity of genes related to starch degradation has, however, rarely been investigated. Based on the kiwifruit genome sequence and previously reported starch degradation-related genes, 17 novel genes were isolated and the relationship between their expression and starch degradation was examined using two sets of materials: ethylene-treated (100 µL/L, 20 °C; ETH) vs. control (20 °C; CK) and controlled atmosphere stored (CA, 5% CO_2_ + 2% O_2_, 0 °C) vs. normal atmosphere in cold storage (NA, 0 °C). Physiological analysis indicated that ETH accelerated starch degradation and increased soluble solids content (SSC) and soluble sugars (glucose, fructose and sucrose), while CA inhibited starch reduction compared with NA. Using these materials, expression patterns of 24 genes that may contribute to starch degradation (seven previously reported and 17 newly isolated) were analyzed. Among the 24 genes, *AdAMY1*, *AdAGL3* and *AdBAM3.1/3L/9* were significantly induced by ETH and positively correlated with starch degradation. Furthermore, these five genes were also inhibited by CA, conforming the likely involvement of these genes in starch degradation. Thus, the present study has identified the genes with potential for involvement in starch degradation in postharvest kiwifruit, which will be useful for understanding the regulation of kiwifruit starch content and metabolism.

## 1. Introduction

Starch is widely distributed in plants and accumulates in various organs, such as leaves, seeds and also tubers [1]. Starch is the major storable metabolite in many plants, and starch degradation is important in plant growth, especially at nighttime [2,3]. *Arabidopsis* mutants that synthesize less starch during the daytime and have lower starch degradation capacity at nighttime exhibited reduced growth rates [4,5]. In fruit, starch mainly accumulates during early fruit development, such as tomatoes [6] and apples [7]. During fruit development, starch is degraded to soluble sugars. In contrast, some fruit, such as kiwifruit [8], mangoes [9], and bananas [10], store a large amount of starch, even in commercially mature fruit, and the starch content can reach approximately 40% of dry matter in mature kiwifruit [11]. High starch-containing fruit such as kiwifruit and bananas degrade starch to soluble sugars, once commercially harvested from vines [12]. Thus, exploration of the mechanisms of starch degradation in fruit would benefit the understanding of the postharvest ripening of kiwifruit.

Multiple enzymes have been reported to be involved in starch degradation, such as glucan, water dikinase (GWD, Enzyme Commission (EC) 2.7.9.4), α-amylase (AMY, EC 3.2.1.1), β-amylase (BAM, EC 3.2.1.2), α-glucosidase (AGL, EC 3.2.1.20), pullulanase (PU, EC 3.2.1.41), isoamylase (ISA, EC 3.2.1.68), α-glucan phosphorylase (PHS, EC 2.4.1.1) and 4-α-glucanotransferase (DPE, EC 2.4.1.25). However, the key regulators of starch degradation vary between different plants, organs and even developmental stages. For instance, GWD was involved in starch degradation in tubers and leaves of potato (*Solanum tuberosum* L.) plants [13,14], while the activity changes in AMY, BAM and AGL suggested that they were associated with starch degradation in postharvest potatoes [15]. Another well investigated model for starch metabolism is *Arabidopsis*, where analysis of transgenic or mutants has shown that *GWD1*, *DPE1* and *DPE2* are involved in starch degradation in leaves [16,17,18], while *AMY1*, *AMY2*, *AMY3* and *PHS1* had limited effects [19,20]. However, it is worth emphasizing that some of the enzymes showed dual functions of starch synthesis and degradation, such as that more than 70% *GWD* reduction could inhibit starch synthesis under light [21], and *GWD* was also involved in starch granule morphology formation [22].

Unlike model plants (e.g., *Arabidopsis*) and staple crops (e.g., rice and potatoes), starch research in fruit, mainly bananas and kiwifruit, has usually only been conducted at the physiological and biochemical level. During the ripening of bananas, activities of AMY, BAM and AGL increased and showed close correlation with starch degradation [23,24], whereas the activities of ISA, PU and PHS were maintained at a relatively constant low level [25,26], and, in kiwifruit, amylase and α-amylase activities have been studied in relation to starch metabolism [27,28]. Only a very limited number of genes related to starch degradation have been studied in postharvest fruit, such as banana *MAmy*, *Ma-bmy*, *Maisa* [10,25], *phoI* and *phoII* [26]. Among the five banana genes, *Ma-bmy* showed increasing expression patterns during postharvest storage [10]. In kiwifruit, several starch related genes (*AdAMY1-3*, *AdBAM1*, *AdBAM3.1*, *AdBAM3.2* and *AdBAM9*) were reported using a kiwifruit expressed sequence tags (EST) database [29]; however, their expression was only analyzed during kiwifruit development (starch accumulation), but not postharvest storage (starch degradation). Thus, the molecular studies on postharvest fruit starch degradation are lacking.

Moreover, the mechanisms of starch degradation in fruit (at least kiwifruit) and model plants (such as *Arabidopsis* leaves) are likely to differ, as starch is degraded to maltose, glucose and glucose 1-phosphate in *Arabidopsis* leaves, but could not be converted to maltose in kiwifruit [30]. Thus, an investigation of starch degradation in kiwifruit would not only benefit our understanding of fruit softening and flavor, but also reveal possible differences in starch degradation in fruit and model plants.

In the present research, changes in starch, fructose, glucose, and sucrose were measured in postharvest kiwifruit. Ethylene treatment and CA storage (vs. normal atmosphere in cold storage, NA) were applied to manipulate kiwifruit postharvest ripening and softening. Using the kiwifruit genome database, genes that encoded *AMY*, *BAM*, *ISA*, *PU*, *GWD*, *DPE*, *PHS* and *AGL* were isolated and analyzed in response to various treatments.

## 2. Results

### 2.1. Isolation and Analysis of Starch-Related Genes

**Figure 1:**
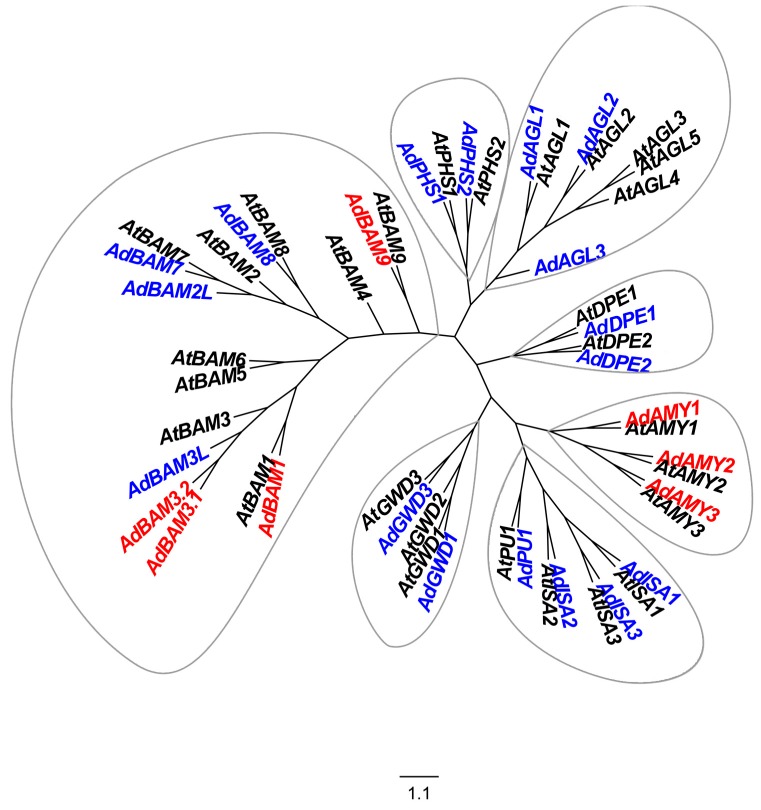
Phylogenetic analyses of starch-related genes from kiwifruit and *Arabidopsis*. Kiwifruit genes are highlighted in **red** (previously reported) and **blue** (newly isolated in the present research). The genes in black were the starch-related genes from *Arabidopsis*. The amino acid sequences of *Arabidopsis* genes were obtained from The *Arabidopsis* Information Resource or National Center for Biotechnology Information. The phylogenetic tree was constructed with Figtree (version 3.1, University of Edinburgh, Edinburgh, UK).

Three *AdAMY* (*AdAMY1-3*) and four *AdBAM* (*AdBAM1*, *AdBAM3.1*, *AdBAM3.2* and *AdBAM9*) genes were reported and analyzed for starch metabolism during kiwifruit development [29]. In addition to these seven known genes, seventeen genes encoding enzymes potentially related to starch degradation were isolated, using the kiwifruit genome database (Figure 1). Based on a phylogenetic tree constructed with *Arabidopsis* homologs and the known kiwifruit genes, these seventeen newly isolated genes were designed as three *AdAGL* (*AdAGL1*-*3*; KX383662-4), four *AdBAM* (*AdBAM2L*, *AdBAM3L*, *AdBAM7*, *AdBAM8*; KX383648-51), three *AdISA* (*AdISA1-3*; KX383653-5), two *AdDPE* (*AdDPE1-2*; KX383660-1), *AdGWD* (*AdGWD1*, *AdGWD3*; KX383658-9) and *AdPHS* (*AdPHS1-2*; KX383656-7), and one *AdPU* (*AdPU1*; KX383652) (Figure 1).

### 2.2. Starch Degradation during Kiwifruit Ripening and Softening at 20 °C

**Figure 2:**
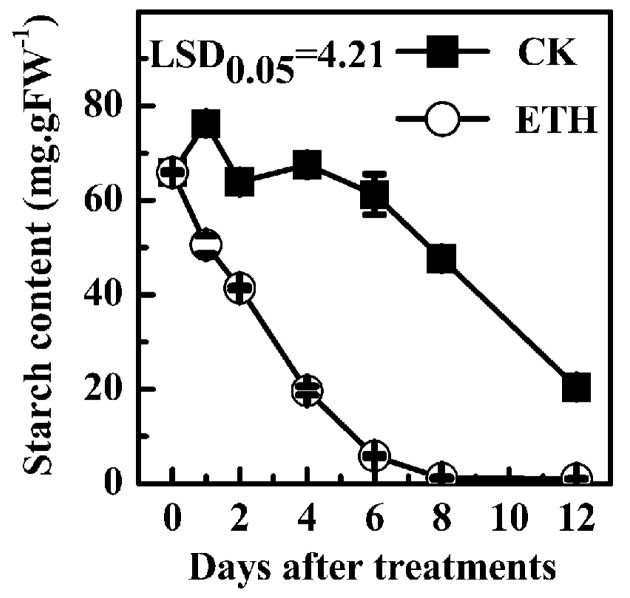
Effect of ethylene treatment (ETH, 100 µL/L, 24 h) on starch content in “Hayward” kiwifruit at 20 °C. CK, the control at 20 °C. Error bars represent ±strandard errors (SE) from three replicates. FW, fresh weight. Least significant difference (LSDs) represent least significant difference at *p* = 0.05.

Kiwifruit (*A. deliciosa* “Hayward”) soften after harvest during storage at 20 °C and ethylene treatment (ETH) rapidly accelerates the fruit softening progress [31]. In parallel with fruit softening, starch content decreased during storage (Figure S1). In control fruit, starch content decreased from 65.9 mg/g at 0 day to 20.4 mg/g at 12 days, while starch content in ETH treated fruit more rapidly decreased and reached the basal level of 1.0 mg/g at 8 days (Figure 2).

**Figure 3:**
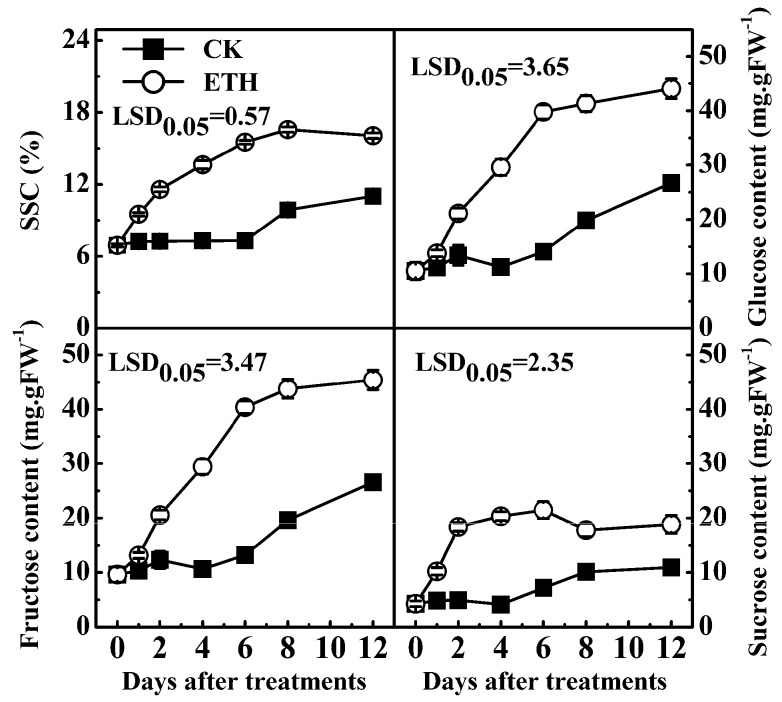
Effect of ethylene treatment (ETH, 100 µL/L, 24 h) on soluble solids content (SSC) and soluble sugars in “Hayward” kiwifruit at 20 °C. Error bars represent ±SE from three replicates. FW, fresh weight. LSDs represent least significant difference at *p* = 0.05.

SSC and contents of soluble sugars were negatively correlated with starch content in kiwifruit (Figure S2). During 20 °C storage, SSC, fructose, glucose and sucrose all showed similar increasing trends, e.g., SSC increased from 6.9% at 0 day to 11.5% at 12 days; fructose, glucose and sucrose increased from 9.6, 10.5, 4.3 to 26.6, 26.7, 10.9 mg/g, respectively, within 12 days of storage (Figure 3). ETH significantly accelerated the accumulation of SSC and soluble sugars, which reached 16.1% (SSC), 45.4 mg/g (fructose), 44.1 mg/g (glucose) and 18.8 mg/g (sucrose), at 12 days (Figure 3).

### 2.3. Expression of Kiwifruit Starch Related Genes in Response to Ethylene Treatment

**Figure 4:**
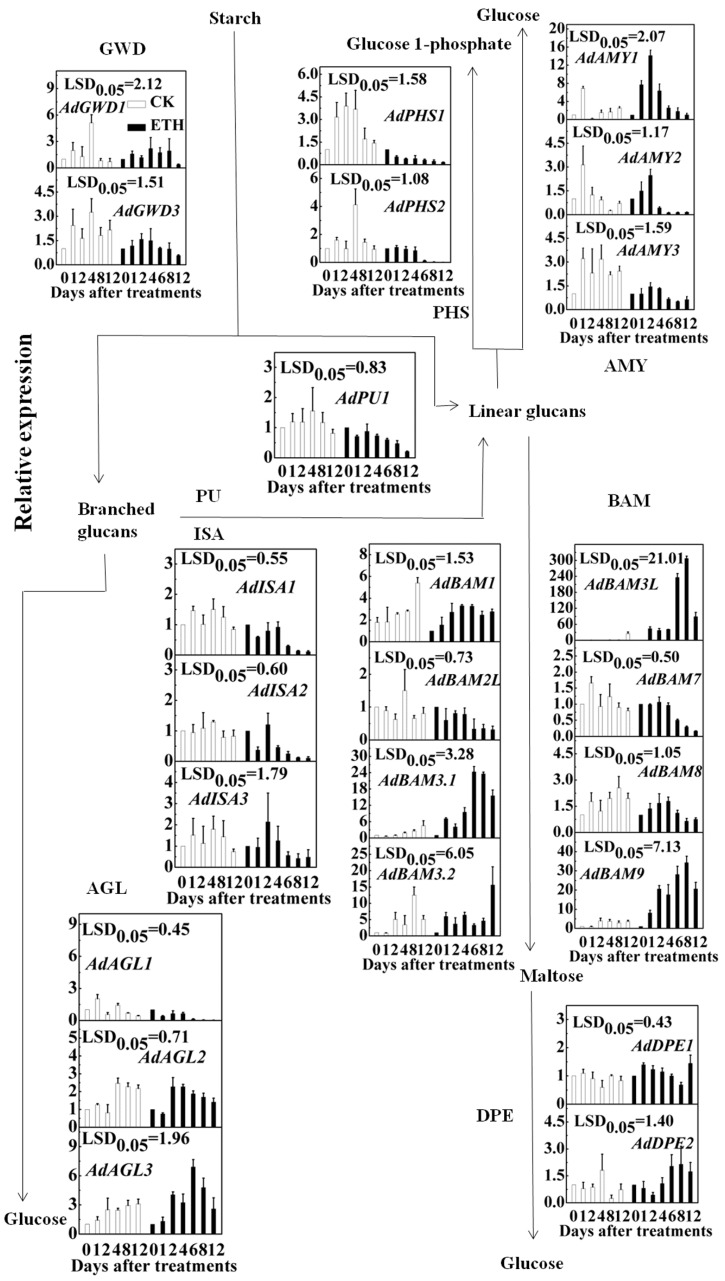
Expression of starch-related genes in response to ethylene treatment (ETH, 100 µL/L, 24 h) at 20 °C. Relative mRNA abundance was evaluated by real-time PCR. White columns, CK; Black columns, ETH. Day 0 fruit values were set as 1. Error bars represent ±SE from three replicates. LSDs represent least significant difference at *p* = 0.05.

Although *AdAMY1-3*, *AdBAM1*, *AdBAM3.1*, *AdBAM3.2* and *AdBAM9* were previously isolated by Nardozza et al. [29], their expression was not analyzed in postharvest kiwifruit. Thus, changes in mRNAs for all newly isolated and previously reported genes were measured in ripening kiwifruit. In control fruit, transcripts from most of these genes increased in abundance, while some others, such as *AdGWD1/3*, *AdAGL1*/*2*, *AdBAM2L/7*, *AdDPE1/2*, *AdISA1-3* and *AdPU1* showed less than a two-fold change (Figure 4). Furthermore, comparison of gene expression in response to ETH and CK indicated that accumulation of transcripts from at least five genes, including *AdAMY1*, *AdAGL3*, *AdBAM3.1*, *AdBAM3L* and *AdBAM9*, was induced by ETH (Figure 4), with *AdBMY3L* being the most responsive, showing an approximately 300-fold increase (Figure 4).

Some of these genes, such as *AdGWD1/3*, *AdAMY3*, *AdPHS1/2* and *AdISA1/2*, were significantly inhibited by ETH (Figure 4). As lower expression of these genes in ethylene-treated fruit is opposite to the desired patterns, these genes were not included for further analysis.

### 2.4. Effect of CA Storage on Starch Degradation and Related Genes in Kiwifruit at 0 °C

**Figure 5:**
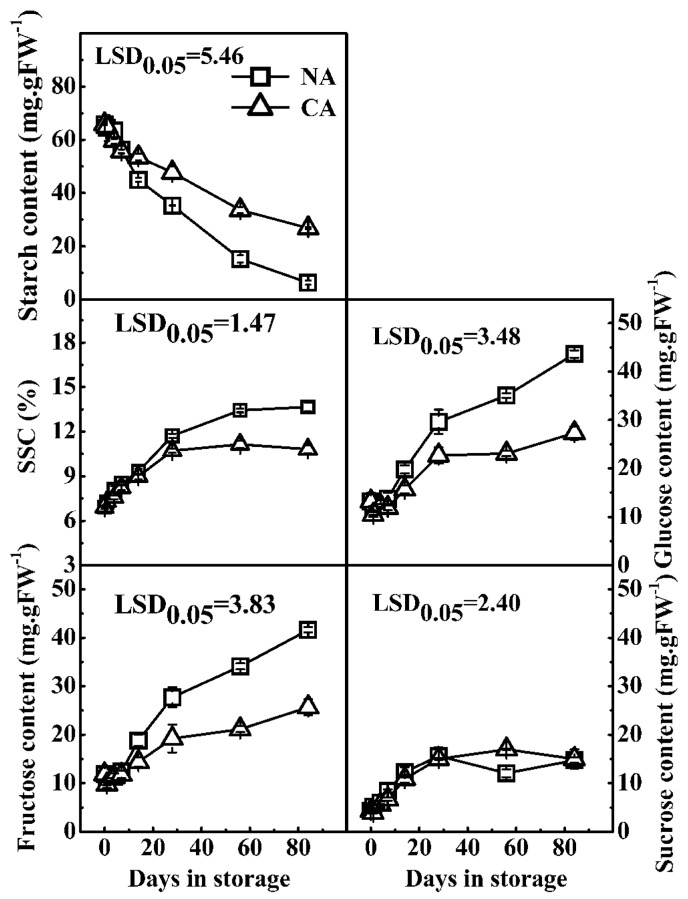
Effect of controlled atmosphere storage (CA, 5% CO_2_ + 2% O_2_) on starch, SSC, and soluble sugars in “Hayward” kiwifruit at 0 °C. NA, normal atmosphere in cold storage (0 °C). FW, fresh weight. Error bars represent ±SE from three replicates. LSDs represent least significant difference at *p* = 0.05.

In order to further analyze the relationship between gene expression and starch degradation, transcripts in fruit held in CA storage and NA at 0 °C were compared. As shown in Figure 5, the starch content was higher in CA treated fruit compared to fruit receiving only cold treatment, which parallels the effect on fruit softening [31]. In contrast, SSC and the content of fructose and glucose increased in postharvest kiwifruit, whereas this was reduced in CA stored kiwifruit, with fructose and glucose content of 25.7 mg/g and 27.3 mg/g at 84 days, compared with control fruit of 41.6 mg/g and 43.6 mg/g, respectively (Figure 5).

**Figure 6:**
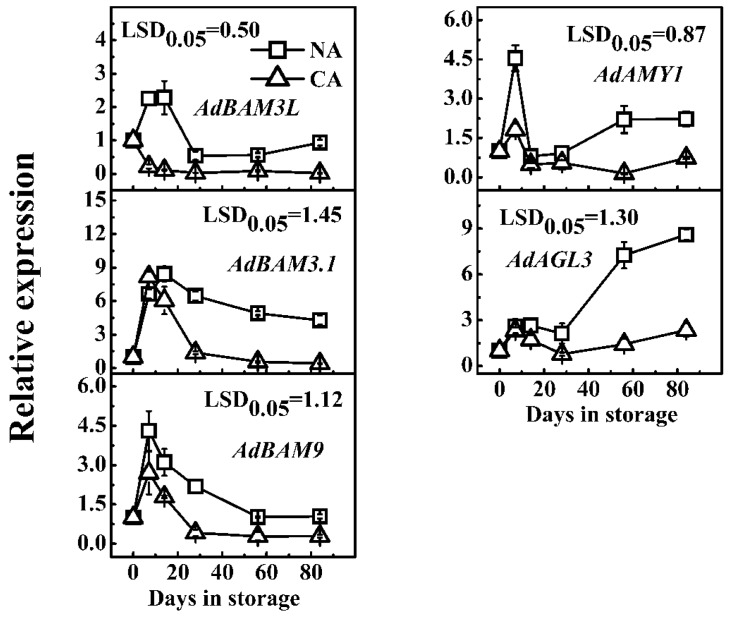
Expression of starch degradation-related genes in response to CA storage (5% CO_2_ + 2% O_2_) at 0 °C. NA, normal atmosphere in cold storage (0 °C). Relative mRNA abundance was evaluated by real-time PCR. Day 0 fruit values were set as 1. Error bars represent ±SE from three replicates. LSDs represent least significant difference at *p* = 0.05.

Here, only the five ETH-induced genes were further analyzed in relation to starch degradation in CA and NA fruit. Generally, transcript abundance of the five genes were higher in NA fruit than those in CA storage (Figure 6). Thus, based on expression patterns, these genes can be divided into two groups, with *AdBAM3L/9* and *AdAMY1* showing significant differences between NA and CA storage at early stages (7 days), while differences in transcript abundance of *AdBAM3.1* and *AdAGL3* mainly occurred at a later stage (56 days and afterwards) (Figure 6).

## 3. Discussion

Maintenance of texture is important for postharvest fruit storability and transportability. Most fruit undergo softening when detached from the tree, resulting from cell wall degradation and reduction in intracellular adhesion [32]. In some fruit, such as kiwifruit and bananas, starch degradation generally happened at initial stages of postharvest softening [12]. This postharvest starch degradation in kiwifruit and bananas is unique behavior, since in many other fruit, conversion of starch to sugars occurs during fruit development. Here, postharvest starch degradation was also observed in Hayward kiwifruit, which was significantly accelerated by ethylene treatment and occurred concomitantly with rapid fruit softening [31]. These results were similar to previous reports [28]. It could be argued that the role of starch degradation for kiwifruit softening, which was mainly considered with contributions of cell wall metabolism. However, the large amount of insoluble starch conversion to soluble sugars may also contribute to fruit softening. In this report, the starch degradation rate was significantly inhibited by CA storage and accelerated by ethylene treatment, and thus both treatments indicated the association of starch degradation and fruit softening.

Despite the importance of starch degradation to both softening and flavor of postharvest kiwifruit, starch-related enzymes and genes have only been studied during kiwifruit development and rarely during postharvest softening. Here, 24 genes potentially related to starch degradation were isolated based on the kiwifruit genome, including 17 novel genes and seven previously studied in developing kiwifruit [29] were investigated during postharvest softening. Although additional genes related to starch degradation were found in the kiwifruit genome, those only expressed in vegetative organs, such as leaves, but not fruit (data not shown) were discarded at the outset.

Taking the results from ethylene treatment (accelerate fruit softening and starch degradation) and CA storage (inhibit fruit softening and starch degradation) together, five genes, including *AdAMY1*, *AdAGL3* and *AdBAM3.1/3L/9*, were identified as having the greatest potential for involvement in starch degradation in postharvest kiwifruit. As shown in Figure 4, these five genes encoded enzymes, AMY, AGL and BAM, which are important for catalyzing conversion of branched and linear glucans to glucose and maltose. Comparing the present kiwifruit results with the literature on bananas [10,23,24] indicates a potential involvement of AMY, AGL and BAM in postharvest starch degradation in both fruit, whereas only *Ma-bmy* was reported to be associated with banana fruit starch degradation according to transcript accumulation [10], and the way is now clear for more genes to be characterized, using transcriptomic analysis of banana genome sequences. The present results and existing literature indicates marked differences in genes involved in starch degradation for different plants. For instance, *GWD* and *DPE* are necessary in potato and *Arabidopsis*, but their transcript levels were quite low during the major starch degradation process of kiwifruit. On the other hand, AMY showed the potential to regulate both banana and kiwifruit starch degradation, which may suggest some overlap mechanisms between different fruit.

## 4. Materials and Methods

### 4.1. Plant Material and Treatments

Mature kiwifruit (*Actinidia deliciosa* (A. Chev.) C.F. Liang and A.R. Ferguson var. *deliciosa* “Hayward”) were chosen as experimental material and were collected and described in our previous report [31]. In brief, the treatments were: ethylene treatment (ETH; 100 µL·L^−1^ C_2_H_4_, 24 h, 20 °C) vs. control (CK; without treatment at 20 °C) and controlled atmosphere (CA; 5% CO_2_ + 2% O_2_, 0 °C) vs. NA (0 °C).

### 4.2. Soluble Solids Content (SSC), Starch and Soluble Sugar Measurements

Meanwhile, SSC were measured at each sampling point. Two ends of each fruit used for firmness measurement were sliced and squeezed. The combined fruit juices from the two slices were measured by an Atago digital refractometer (Tokyo, Japan). SSC measurements were conducted with 12 fruit replicates, with 4 fruit from each biological replicate. After SSC analysis, as well as firmness analysis [31], the outer pericarp of 12 fruit were collected, according to the three biological replicates. These samples were frozen in liquid nitrogen and stored at −80 °C for further use, such as starch and soluble sugar measurement and RNA extraction.

Starch and soluble sugar (fructose, glucose, sucrose) measurements were conducted with frozen materials described above in three biological replicates. Total starch was extracted from 0.1 g frozen samples for each replicate following the procedures of the total starch assay kit (Megazyme International Ireland Ltd., Wicklow, Ireland), as described by Stevenson et al. [11]. The absorbance for each sample and the d-glucose control were read at 510 nm against the reagent blank with UV2550 (Shimadzu, Kyoto, Japan). Glucose, fructose and sucrose extractions and measurements were conducted according to the protocol of Lisec et al. [33]. Soluble sugars were extracted from 0.1 g frozen samples, which were incubated with 1.4 mL methanol (100%) at 70 °C for 15 min in a Thermomixer (Eppendorf, Hamburg, Germany) at 950 rpm. After centrifuge 11,000× *g* for 10 min, the supernatants were transferred to new tubes and were mixed with 750 µL chloroform and 1400 µL dH_2_O. Then, the mixtures were centrifuged at 2200× *g* for 15 min. Aliquots of 100 µL of the supernatants were dried in vacuum (Eppendorf, Hamburg, Germany) and 20 µL ribitol (0.2 mg·mL^−1^) was included in each sample as an internal standard. The residue was dissolved in 40 µL of 20 mg/mL pyridine methoxyamine hydrochloride, and incubated for 1.5 h at 37 °C. The sample was then treated with 60 µL Bis (trimethylsilyl) trifluoroacetamide (1% trimethylchlorosilane) for 30 min at 37 °C. The soluble sugars were measured by GC (Agilent Technologies 7890A, Santa Clara, CA, USA), A volume of 1 µL for each sample was absorbed with a split ratio 10:1 and injected into the gas chromatograph fitted with a fused-silica capillary column (30 m × 0.25 mm i.d., 0.25 µm DB-5 MS stationary phase). The injector temperature was 250 °C and the helium carrier gas had a flow rate of 1.0 mL/min. The column temperature was held at 100 °C for 1 min, increased to 184 °C with a temperature gradient of 3 °C/min, increased to 190 °C at 0.5 °C/min, held for 1 min, increased to 280 °C at 15 °C/min and then held for 3 min. The significant MS operating parameters were as follows: ionization voltage was 70 eV, ion source temperature was 230 °C and the interface temperature was 280 °C.

### 4.3. RNA Extraction and cDNA Synthesis

Total RNA extractions were conducted according to the method described by Yin et al. [34]. The genomic DNA in total RNA was degraded with TURBO Dnase (Ambion, Life Technologies, Gaithersburg, MD, USA). cDNA was synthesized from DNA-free RNA, using reverse Transcription System (Promega, Madison, WI, USA). RNA extractions and cDNA synthesis were conducted with three biological replicates for each treatment at each sampling point.

### 4.4. Gene Isolation and Analysis

Starch degradation-related genes mentioned in research on starch metabolism during kiwifruit development, such as α-amylase genes (*AdAMY1-3*) and β-amylase genes (*AdBAM1*, *AdBAM3.1*, *AdBAM3.2* and *AdBAM9*) [29], were selected for analysis in relation to postharvest starch degradation in ‘Hayward’ kiwifruit. Furthermore, using the kiwifruit genome database [35], additional starch degradation-related genes were isolated, with primers listed in Table S1.

Alignment was performed using the neighbor-joining (NJ) method in ClustalX (v. 1.81, University College Dublin, Dublin, Ireland) and phylogenetic analysis was performed with online software Figtree (v 3.1, University of Edinburgh, Edinburgh, UK; Available online: http://tree.bio.ed.ac.uk/software/figtree/). Accession numbers for *Arabidopsis* genes in The Arabidopsis Information Resource (TAIR) are as follows: *AtAMY1*-3 (Genbank no. At4g25000, At1g76130, At1g69830); *AtBAM1-9* (Genbank no. At3g23920, At4g00490, At4g17090, At5g55700, At4g15210, At2g32290, At2g45880, At5g45300, At5g18670); *AtISA1*-*3* (Genbank no. At2g39930, At1g03310, At4g09020); *AtPU1* (Genbank no. At5g04360); *AtPHS1-2* (Genbank no. At3g29320, At3g46970); *AtGWD1-3* (Genbank no. At1g10760, At4g24450, At5g26570); *AtDPE1-2* (Genbank no. At5g64860, At2g40840); and *AtAGL1-5* (Genbank no. At3g23640, At5g63840, At3g45940, At5g11720, At1g68560).

### 4.5. Real-Time PCR Analysis

For real-time PCR, all primers were designed with online software primer3 (v. 0.4.0, available online: http://frodo.wi.mit.edu/cgi-bin/primer3/primer3_www.cgi) and are listed in Table S2. Gene specificity of each pair of primers was confirmed with PCR product resequencing [36]. *AdACT* was chosen as the internal control [36]. Gene expression was analyzed using a LightCycler480 instrument (Roche, Berlin, Germany), with the same PCR programs described by Zhang et al. [31].

### 4.6. Statistical Analysis

Least significant difference (LSD) was calculated with DPS (v7.05, Zhejiang University, Hangzhou, China). Figures were prepared with Origin 8.0 (Microcal Software Inc., Northampton, MA, USA).

## 5. Conclusions

In conclusion, seventeen genes encoding enzymes related with starch degradation were newly identified and isolated based on the kiwifruit genome, expanded in this catalog in kiwifruit from seven [29] to 24 genes. Among them, five genes, *AdAMY1*, *AdAGL3* and *AdBAM3.1/3L/9*, were characterized, using various postharvest treatments, as having higher potential to be involved in starch degradation during kiwifruit postharvest ripening. This has provided more specific targets for determining the mechanism and regulation of starch metabolism in kiwifruit. The potential complexity, however, with the involvement of multiple genes, may increase the difficulty for modification by transgenic or classical breeding approaches. Thus, it will be important to find the master regulators (e.g., transcription factors) for these five genes in future studies.

## Supplementary Materials

Supplementary materials can be found at www.mdpi.com/1422-0067/17/12/2112/s1.

## Abbreviations

AMY	α-Amylase
BAM	β-Amylase
CA	Controlled atmosphere
ETH	Ethylene
ISA	Isoamlase
GWD	Glucan water dikinase
PHS	Glucan phosphorylase
DPE	4-α-Glucanotransferase
PU	Pullulanase
AGL	α-Glucosidase
SSC	Soluble solids content
GC	Gas chromatography
