# Supplementary material for: Characterization of Starch Degradation Related Genes in Postharvest Kiwifruit
Source: Int J Mol Sci. 2016 Dec 15;17(12):2112. doi: 10.3390/ijms17122112 (PMC5187912; doi:10.3390/ijms17122112)
Supplement: Supplementary file 1 [file ijms-17-02112-s001.pdf]

Supplementary Material: Characterization of Starch Degradation Related Genes in Postharvest Kiwifruit

Xiong Hu, Sheng Kuang, Ai-Di Zhang, Wang-Shu Zhang, Miao-Jin Chen, Xue-Ren Yin and Kun-Song Chen

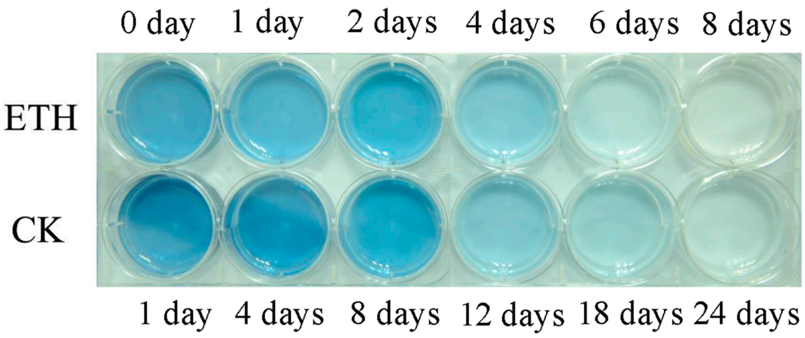

Figure S1. Staining of extracted starch from kiwifruit. Extraction of starch was conducted according to the method described by Stevenson et al. [11] and the **blue** color was indicated by I_2-KI. Intensity of the **blue** color indicated the starch content.

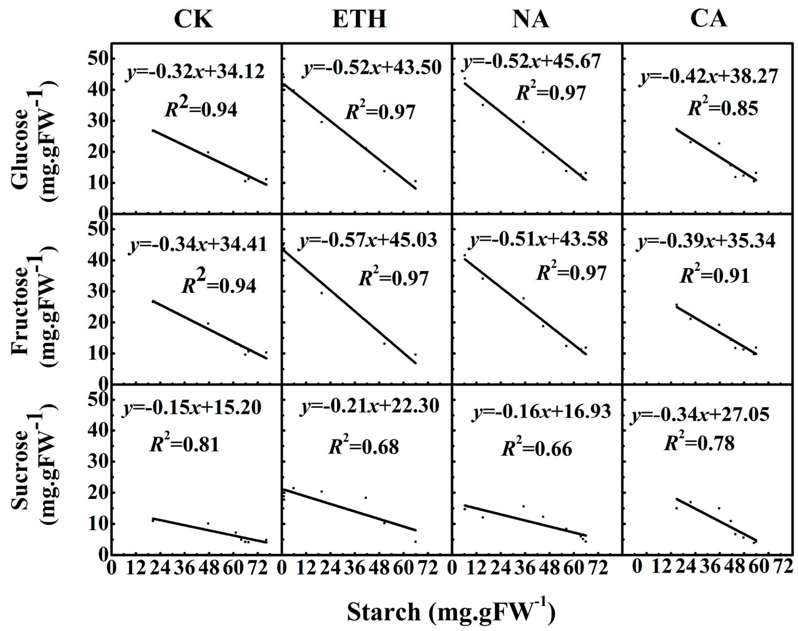

Figure S2. Linear correlation analysis between starch content and soluble sugars. The data used in this figure are same to Figures 2, 3 and 5 in the manuscript. R^2 values were calculated with Microsoft Excel.

Table S1. Primers used for gene amplification.

Gene	Forward Primer (5′–3′)	Reverse Primer (5′–3′)
AdAMY1	ATGACCACCAATTTTCTCAGCTTCG	CGATCTTGGAAACCTTATTCCACCTAA
AdAMY2	ATGACCGTTAACATTCCCTCCG	GATACGCAGTATGGCACAAGTAA
AdAMY3	ATGCCGACAGTGACTCTAGAGCCG	GCAGCACATGTTCACCTTGTTTGA
AdBAM1	ATGACTACGGAGATGCAGAGATTTGG	CTGCTGGAGGTGGGGAGAAGAAGAAGTGA
AdBAM2L	ATGGCGATTTCGTCCGCTAAACTGTTC	CTCCCTCTAACGAAAAGGGACTGA
AdBAM3.1	ATGGCTTTGACGCTACGTTCTTCAAC	GATGAAGAAGGCCGCTCTAGTGTAA
AdBAM3.2	ATGGCTTTGACGCTACGTTC	TGAATAAGAGGTTGTTCGAG
AdBAM3L	ATGGCTTTAACATTACATTGTTCG	AGGAGGCTGCTTTTGTGTAA
AdBAM7	ATGACTACGGAGATGCAGAGATTTGG	CTGCTGGAGGTGGGGAGAAGAAGAAGTGA
AdBAM8	ATGAACCACCACCCGAACCAAGATC	GTTAAATCCATGCATGGTATGACCTGA
AdBAM9	ATGGAGGTTTCGGTGATCGGA	CTCCACATGCAAGTTGCTTAG
AdISA1	ATGGCGTTACTTCAGTATATATCGT	CTGGTGATGGTGATGGTGCTTAGTT
AdISA2	ATGGCAACTCTTCCACTATC	GCCAAGAAACTAAGTGATTGA
AdISA3	ATGTTCGTAGATGCTCTGCTTTCAT	GATTCTTCTGGAGGCGAAGCCATGA
AdPU1	ATGTCTCTGTTACGATTATCACCATC	GTGTTTGTTGAGTCTAGAAACATCTGA
AdPHS1	ATGGCGACTTCAAGGTTCTCTGCGC	GAACATTAAACCCGTTGCATTACCATAG
AdPHS2	ATGGCTACTACAGCAGAAGCC	AGGAGTGCCGCGTACCATAA
AdDPE1	ATGGATCTCATCTCATCCAC	GTCAGGGTTCATTTTGACTT
AdDPE2	ATGGTCAACTATAGTTTGAT	GAAATTTTAGGATGTATTGC
AdGWD1	ATGAGCAATTCGATAGGGCATAACTTTC	GACCCCAGATGTAATATTGATTCTCTC
AdGWD3	ATGGATGCTCTGCGTGTATT	AGTTCGTGCTGCTATTCATT
AdAGL1	ATGTCTGAGTTTGAAGATGCGGCAG	GTACGAGGTGAGGGGAATCTCGTAG
AdAGL2	ATGAGAAGTGTATCTCTGTATCATC	CAATTGGACGATTCAAGTTTTATAG
AdAGL3	ATGGAAACTCTCAAAATCACCAAAAAG	TATTGTAAAGTAAAGGATTACCCATC

Table S2. Primers used for real-time PCR.

Gene	Enzyme	Forward Primer (5′–3′)	Reverse Primer (5′–3′)
AdAMY1	α-amylase	CCTCTGGCAATGACTATGCTG	TAGTAATTGCCCGTGCCC
AdAMY2		TTCACGCTGATAGGTTAATGG	CAAGGCATCTATCTGTTATGCC
AdAMY3		GGAAGTGATCATGTCCACGTAA	CAGAGGCAAGAGGGTATTGTAA
AdBAM1	β-amlase	CTTGGCCTTTGGTACAGGAA	CTCCTCCATGGCTTTTATGTG
AdBAM2L		CGCAATTATGACATGGTTCCT	GTTTATTTTGGTGGGAGGCA
AdBAM3.1		TCGAGCAGAACGGACCTTTA	GCTGAGTTTTGAAGATGGCTG
AdBAM3.2		CCAAGTGGAGTTTGCCCCTT	GGAAGATGAAGGAGGCTGCT
AdBAM3L		GTATATATTATATGTGCATGTTC	GAAGCTGACAAAAGCTCATT
AdBAM7		ACGGGGAAGCAGTTCTTGAT	TAGCACAATCCACTGCTTGG
AdBAM8		AATTGCAGGTGACCGAGAAC	TACCCCATAGACTTGGGTGG
AdBAM9		CTCCACATGCAAGTTGCTTAG	CCTAATCTTCCTTGGAAGC
AdPU1	pullulanase	CCCAAGGACGACTTCTGTGT	AGGTGAAACGATTGTGAGGG
AdISA1	isoamylase	GTTCCTCGAGTGATGCCATT	AAATAGAAATCCGAGCACCG
AdISA2		GCCAAGAAACTAAGTGATTGA	GGATCTATTTTGGAAGGT
AdISA3		TCTCGAGTCTCCTGGTGATA	GGAACCTTTCCCTAACAGTATC
AdPHS1	α-glucan phosphorylase	TGGTGCTCAAGCTCAAGAGA	CTATTTCCTTGTGGGCAAGG
AdPHS2		ACTGCCACCCCAATCCCAT	ACTGTATTTGCACTGTGCAG
AdGWD1	α-glucan water dikinase	GTTGCGCTTGATTATTCATC	TAGACCTCTGTTCCTGGTTC
AdGWD3		CTTGAGAGGTGAATTTCATG	AGTTCGTGCTGCTATTCATT
AdDPE1	4-α-glucanotransferase	GTACATGCTGATATTTTCGC	GTCAGGGTTCATTTTGACTT
AdDPE2		AGCATCAATTTCGTACCGAG	GAAATTTTAGGATGTATTGC
AdAGL1	α-glucosidase	CCTGTTTCGAAGCAATCGCC	CGGCTCTGAATGTCGAAATGG
AdAGL2		AATAAATGGAAGGGCGGCT	TTCTACAGTGCTTCAATGGGC
AdAGL3		GGTTCTGAAGTTTTGGTGGA	GCATGTACATTCCAATAATC
